# A gap and synergy analysis of the European research infrastructure (RI) ecosystem: advancing the novel GRACE-RI dedicated to plant genetic resources

**DOI:** 10.1093/aob/mcaf092

**Published:** 2025-06-10

**Authors:** Domenico De Paola, Francesca Taranto, Soraya Mousavi, Francesco Mercati, Wilma Sabetta, Marina Tumolo, Sharif Islam, Roland Pieruschka, Andrea Scaloni, Anne-Francoise Adam-Blondon, Lorenzo Maggioni, Sandra Goritschnig, Filippo Guzzon, Massimo Ianigro, Giovanni Giuseppe Vendramin, Giovanni Giuliano, Gabriele Bucci

**Affiliations:** CNR-IBBR Institute of Biosciences and Bioresources, National Research Council of Italy, Bari 70126, Italy; CNR-IBBR Institute of Biosciences and Bioresources, National Research Council of Italy, Bari 70126, Italy; CNR-IBBR Institute of Biosciences and Bioresources, National Research Council of Italy, Perugia 06128, Italy; CNR-IBBR Institute of Biosciences and Bioresources, National Research Council of Italy, Palermo 90146, Italy; CNR-IBBR Institute of Biosciences and Bioresources, National Research Council of Italy, Bari 70126, Italy; CNR-IBBR Institute of Biosciences and Bioresources, National Research Council of Italy, Bari 70126, Italy; Naturalis Biodiversity Centre, Leiden 2333 CR, The Netherlands; DiSSCo- Infrastructure for Distributed System of Scientific Collections; IBG-2 Plant Sciences, Forschungszentrum Jülich, Jülich 52428, Germany; EMPHASIS-European Infrastructure for Plant Phenotyping; CNR-ISPAAM, National Research Council of Italy, Portici 80055, Italy; METROFOOD- Infrastructure for Promoting Metrology in Food and Nutrition; Université Paris-Saclay, INRAE, URGI, French Network of Biological Resource Centres for Research in Biology, Agronomy and Environment, Versailles 78026, France; ECPGR Secretariat, c/o Alliance of Bioversity International and CIAT, Rome 00153, Italy; ECPGR Secretariat, c/o Alliance of Bioversity International and CIAT, Rome 00153, Italy; ECPGR Secretariat, c/o Alliance of Bioversity International and CIAT, Rome 00153, Italy; CNR-IRSA Water Research Institute, National Research Council of Italy, Bari 70132, Italy; CNR-IBBR Institute of Biosciences and Bioresources, National Research Council of Italy, Sesto Fiorentino 50019, Italy; ENEA National Agency for New Technologies, Energy and Sustainable Economic Development, Casaccia Research Centre, Rome 00123, Italy; CNR-IBBR Institute of Biosciences and Bioresources, National Research Council of Italy, Sesto Fiorentino 50019, Italy

**Keywords:** Crop diversity, ESFRI roadmap, research infrastructure, GRACE-RI, plant genetic resources, *ex situ* and *in situ* PGR conservation, genebanks, gap analysis, synergy analysis

## Abstract

**Background:**

Plant genetic resources (PGRs) are crucial for sustainable agriculture and food security, but the roadmap of the European Strategy Forum on Research Infrastructures (ESFRI) lacks a dedicated research infrastructure (RI) for their systematic cataloguing, safeguarding and improvement. To fill this gap, we propose a new RI concept specifically for PGRs in Europe.

**Scope:**

The proposed RI, called ‘Plant Genetic Resources Community for Europe’ (GRACE), is aimed to support current and future research projects on PGRs, enhance collaboration across European countries, unlock the adaptive potential of crop biodiversity preserved in PGR collections, and strengthen the current and future sustainability of the food chain in Europe. As part of the preparatory project ‘Promoting a Plant Genetic Resource Community for Europe’ (PRO-GRACE), we analysed the current landscape of European RIs supporting PGR-related research in complementary fields regarding research aims, research products and features/services.

**Conclusions:**

Through a robust quantitative approach, we have identified gaps and potential synergies among six RIs from the Health and Food and Environment domains of the ESFRI roadmap. These findings were discussed in the context of European PGR research priorities and current societal needs, and the implementation of GRACE was proposed as a strategic response to these challenges.

## GENERAL OVERVIEW OF PLANT GENETIC RESOURCES IN EUROPE

Plants are essential for life on Earth. Terrestrial plants fix ∼50 % of human-made CO_2_ and produce ∼50 % of the oxygen we breathe, and, directly or indirectly, most of the organic matter that we use as food. There are ∼400 000 terrestrial plant species, and we use ∼6000 of them for food (Pilling *et al*., 2020).

We are living at the beginning of the 6th large mass extinction; terrestrial plant species are going extinct at rates 500 times larger than at pre-industrial levels, with projections bringing this figure as high as 10 000 times in the future (Antonelli *et al*., 2023). Each time a plant species goes extinct, a genepool of tens of thousands of potentially useful genes for both our survival and that of the ecosystems goes extinct with it. Therefore, the preservation of plant biodiversity by conserving plant genetic resources (PGRs) both *in situ* (in their natural environments) and *ex situ* (conserving samples of wild populations and/or crop varieties in specialized structures such as botanical gardens, genebanks or conservation seedbanks) is key to our survival on the planet.

The conservation and sustainable use of PGRs is crucial for ensuring food security in Europe in the next decades (Maxted and Kell, 2021; Swarup *et al*., 2021). Extensive regions of Europe's agricultural landscape are expected to be significantly impacted by the effects of climate change and human activities, increasing losses in biodiversity and habitat degradation (Thuiller *et al*., 2005, 2011; Darbyshire *et al*., 2017; Bajocco *et al*., 2018; Agovino *et al*., 2019; Bhadouria *et al*., 2019). Changes in habitat suitability for crop varieties and wild species can have significant economic and social consequences due to the geographical shift of their cultivation areas (Santos *et al*., 2020; Jägermeyr *et al*., 2021). The wide effects of climate change on European agriculture (Hannah *et al*., 2013, Malhi *et al*., 2021; Skendžić *et al*., 2021) call for a paradigm shift towards more sustainable food production systems, including crop varieties more efficient in nutrient uptake, as well as crop diversification to mitigate the effects of extreme climatic events. Furthermore, the future sustainability of the food system is threatened by the continuous growth of the world’s population: to meet the global food demand by 2050, agricultural productivity must increase by 60 %, without substantially expanding arable land (Silva, 2018).

PGR collections, both *ex situ* and *in situ* (*ex situ* genebanks, on-farm landraces, *in situ* reserves, etc.), are treasure chests of largely unknown functional diversity for many crop species and key sources of relevant genes, and they can significantly contribute to agriculture resilience by enhancing crop adaptations to global change and long-term sustainability of food production (Weise *et al*., 2020; Aubry, 2023). They represent a barrier against the progressive loss of both intra- and inter-specific diversity and genetic erosion that has occurred over the last century, mainly due to the selection of a few high-yielding plant varieties to meet the majority of food needs (McCouch *et al*., 2020; Khoury *et al*., 2022). Therefore, an urgent need is to broaden the genetic base of elite germplasm of plants, for long-term genetic gain and to strengthen resistance to biotic and abiotic stresses. Despite the large adaptive potential of the genetic and phenotypic diversity stored in collections and the non-negligible costs for their preservation, only a small proportion of over 2 million genebank accessions preserved *ex situ* by about 400 institutes and 6384 *in situ* crop wild relative populations from 11 European pilot countries currently listed in the European Search Catalogue for Plant Genetic Resources (EURISCO) (www.ecpgr.org/eurisco; Kotni *et al*., 2023) have been used in pre-breeding/breeding activities to improve commercial varieties or create new ones (McCouch *et al*., 2020; Volk *et al*., 2021; Sanchez *et al*., 2023; Cheng *et al*., 2024). The potential use of genebank materials for the genetic improvement of crop species is hampered by the fact that a large proportion of accessions is poorly characterized, with available data often limited to their geographical origin but lacking genetic, molecular and/or phenotypic information. In addition, the efficiency and overall operational quality of genebanks, the conditions governing the access to these resources and the lack of clearly defined access policies can affect final accession availability (Brink and van Hintum, 2020; Smith *et al*., 2021).

In the last few decades, cutting-edge research has been conducted in the frame of various EU-funded projects on PGRs, such as HARNESSTOM (https://cordis.europa.eu/project/id/101000716/reporting/it), BREEDINGVALUE (https://breedingvalue.eu/), AGENT (https://agent-project.eu/), G2P-SOL (http://www.g2p-sol.eu/), ECOBREED (https://ecobreed.eu/project/), TRADITOM (https://traditom.eu/it/) and INCREASE (https://www.pulsesincrease.eu/). These projects generated a large amount of PGR-related passport data, phenotypic characterization and evaluation (C&E) data as well as multi-omic data stored in different repositories and platforms (König *et al*., 2020; Barchi *et al*., 2021, 2023; Gonzalo *et al*., 2021 ; Tripodi *et al*., 2021; Calle García *et al*., 2022; Mellidou *et al*., 2023; von Steimker *et al*., 2024), but which may not have been regularly maintained after the end of the project, so there is a risk that they will be discontinued with data loss. In addition, the above-reported repositories/platforms often offer different accessibility and usability of data/datasets, and different ontologies/semantic standards are often adopted, which can result in data fragmentation and duplication (Gabdank *et al*., 2018). Such siloed efforts and insufficient data interoperability among platforms ultimately lead into a complex labyrinth that is often difficult to navigate by users, although the application of FAIR principles (Findable, Accessible, Interoperable, Reusable) has gained more prominence in recent years (Wilkinson *et al*., 2016). Additionally, the connection between the plant biological material used or sampled and the data from multi-omic experiments is too often missing (Jamil *et al*., 2020). This hampers the genomic-based prediction of performances of crop species and hinders their selection or use in breeding programmes for the improvement of cultivated varieties (Blätke *et al*., 2021; Aubry, 2023).

Since 1980, the European Cooperative Programme for Plant Genetic Resources (ECPGR, www.ecpgr.org) has been active in promoting the effective conservation and sustainable use of PGRs through a network of national PGR programmes, the development of infrastructures, frameworks and tools, and building capacity and knowledge. In the ECPGR Plant Genetic Resources Strategy for Europe (ECPGR 2021), the establishment of a robust PGR research infrastructure (RI) with a secure and sustainable financial basis is presented as crucial to safeguard Europe’s plant genetic resources for future generations and to contribute to global efforts towards biodiversity conservation and food security.

The above scenario emphasizes the need for a coherent European policy framework and the development of a long-term European RI dedicated to PGRs, encompassing large-scale facilities, digital platforms and essential services (Goritschnig *et al*., 2025).

The European Strategy Forum on Research Infrastructures (ESFRI) supports the development and integration of high-quality RIs across Europe promoting a coordinated approach among EU Member States, Associated Countries and the European Commission. Although PGRs are essential in many research and innovation activities that mobilize genetic diversity and therefore require infrastructure-based approaches for their conservation and access, no RI dedicated to PGRs is currently listed in the ESFRI Roadmap 2021 (https://roadmap2021.esfri.eu/), the EU’s strategic planning document which identified major pan-European RIs of strategic importance. This represents a relevant gap in the European RI landscape, particularly because food security has already been recognized as crucial in the future EU ‘one-health’ scenario (OHEJP 2023).

## PGRs: TURNING CHALLENGES INTO OPPORTUNITIES

Recent advancements in technology have significantly enhanced our ability to generate and analyse big data. This creates an unprecedented opportunity to develop new strategies for conserving and exploiting PGRs, and for promoting their use in agriculture. Furthermore, using advanced sequencing and phenotyping technologies to screen PGR collections would improve the maintenance of crop germplasm, reducing its costs by optimizing basic genebank operations, from prioritizing the regeneration of plant material to identifying gaps or redundancies in the collections (van Treuren and van Hintum, 2014). Some examples of the application of -omic technologies on collections held in genebanks are reported below. Sequence-based genotyping methods can help maximize genetic variability within genebanks through the development of core collections (Jia *et al*., 2017). Genomics can also be used to identify redundancies in collections, as proposed by Singh *et al*. (2019), who assessed genetic diversity in a panel of 1143 accessions of *Aegilops tauschii* Coss., an economically important wheat wild relative, from three genebanks and found an average of about 50 % duplicates, highlighting the importance of optimizing the composition and size of the collection to improve the efficiency of funds spent on conservation. In other cases, such as *Solanum melongena* L. and its relatives, redundancy is much less, ranging between 0.6 and 15 % (Barchi *et al*., 2023). The extent of detected redundancy depends on the sensitivity of the barcoding method adopted, and the need to rely on both molecular methods and phenotypic data to assess redundancy has been stressed (Barchi *et al*., 2023).

To fully exploit the adaptive potential of the phenotypic diversity preserved in genebanks, it is essential to make available phenotypic information about the collections along with the genetic characterization of the material. Linking genotypic and phenotypic data can help exploit core collections tailored to specific traits or facilitate efficient querying of germplasm catalogues to find lines with desired genetic elements, ultimately strengthening the role of genebanks in maintaining PGR genetic diversity useful to breeding (Halewood *et al*., 2018; Wambugu *et al*., 2018; Mascher *et al*., 2019). For example, genome-wide association studies (GWAS) of a global collection of *Capsicum* spp. comprising 10 000 accessions from 130 countries and five continents were carried out to reconstruct the evolutionary history of the species and allowed the identification of several markers associated with important traits under selection (Tripodi *et al*., 2021). Challenges posed to agriculture by environmental changes may be addressed by predicting genotype-by-environment (G × E) interactions through the massive sampling of a genepool across a broad spectrum of environmental conditions, which may lead to the development of crop varieties tailored to specific environmental contexts (Halewood *et al*., 2018; El Hanafi *et al*., 2023), thereby mitigating harmful impacts of climate change as demonstrated with *Triticum aestivum* L. (Fradgley *et al*., 2023) or *Zea mays* L. (Welcker *et al*., 2022). The challenge of characterizing and exploring the large number of accessions held in genebanks requires a concerted effort by a wide range of conservation and research institutions. An effective framework for this type of collaboration is not yet in place. Furthermore, due to limited resources, the efforts of genebanks need to be oriented towards ensuring the long-term conservation and availability of accessions and they sometimes have limited access to facilities and expertise connected to multi-omic technologies (Gupta *et al*., 2020). In addition, the application of -omic technologies requires the development of standardized protocols to ensure the quality and reproducibility of results (Weise *et al*., 2020). In this scenario, better coordination and support for improving characterization, conservation and access to genetic resources is urgently needed. This includes establishing specific standards for PGR management in Europe and, *inter alia*, developing a common, widely accepted system for the certification of genebanks (van Hintum and Wijker, 2024).

Enhancing the use and valorization of PGR collections also requires structured and easily accessible documentation of the preserved germplasm. Weise *et al*. (2020) emphasized the importance of transitioning from the current local management of PGR data to networked/federated remote repositories to ensure broader access to PGR documentation and its safe, long-term storage. Such a transition is essential to prevent the loss of documentation over time due to, for example, changes in future storage technologies and/or the turnover of local personnel managing these collections. To this end, EURISCO represents a successful model of an information system collecting and providing passport data from 43 national collections and ongoing efforts to enhance the coverage of C&E data and information on *in situ* conserved populations. A further step will be the integration of accession documentation systems with databases and data archives such as the EMBL (https://www.ebi.ac.uk/), which are intended for -omic data.

## GRACE-RI: A NEW PILLAR FOR THE PGR COMMUNITY

The PRO-GRACE project consortium (funded by Horizon Europe, https://www.grace-ri.eu/pro-grace) proposes a pan-European research infrastructure named GRACE (Plant Genetic Resources Community for Europe) to strengthen the implementation of *ex situ*, *in situ* and on-farm conservation of PGRs and to establish a broader, coordinated network of conservation centres. *Ex situ* conservation will be supported by a structured framework, with standardized methodologies and coordinated efforts across Europe – most notably through initiatives such as AEGIS (https://www.ecpgr.org/aegis), which standardize the activities of individual genetic resource centres, improving their quality and eventually aiming at a certification system. In contrast to genebanks, *in situ* conservation remains less developed, often relying on fragmented, locally coordinated initiatives, involving a wide variety of stakeholders both at the regulatory and implementation level. Despite these limitations, *in situ* conservation is best suited for preserving the widest genetic diversity of landraces and crop wild relatives, making it an essential component of a comprehensive conservation strategy. GRACE-RI will be dedicated to endowing the European region with an efficiently conserved, well-documented and accessible reservoir of PGRs, maintained in genetic resource centres and in natural habitats, and made available for research and the public good at all times. Moreover, building on existing national conservation programmes and international networks and initiatives, GRACE-RI will develop a range of technological and scientific services to enhance and optimize the long-term conservation of PGRs and promote their accessibility and use in breeding. To this end, the implementation of a Quality Management System (QMS) for the conservation and accessibility of PGRs, in collaboration with AEGIS, aims to address an important gap in the current EU landscape and represent a reference model for European genebanks, providing end users – such as researchers, breeders, farmers and seed companies – with high-quality germplasm materials along with the related data, documentation and knowledge. Safety duplication of unique accessions conserved *ex situ* and *in situ*, as well as reconstruction and maintenance of pedigree information and kinship relationships of the plant materials conserved in genebanks, are additional objectives of GRACE-RI that are largely incomplete or currently lacking in the existing RIs. Moreover, the identification of duplicated accessions and potential gaps in diversity within and between genebanks and *in situ* collections will be coordinated by GRACE-RI at the European level. To this end, standard workflows, procedures and practices for the maintenance and characterization of the experimental material are required, and this is currently a gap in the PGR scenario and in the European RI landscape. Further, the novel infrastructure will promote cooperation with several existing RIs for improving current taxonomic validation services with specific and intraspecific information on crop taxa. Finally, GRACE-RI will provide the PGR community with tools to enhancing access to PGRs, i.e.: (1) advanced protocols for phytosanitary assessment and sanitation; (2) legal services for navigating the international treaties governing PGR exchange, such as the International Treaty on Plant Genetic Resources for Food and Agriculture (https://www.fao.org/plant-treaty/en/) and the Nagoya Protocol on Access and Benefit Sharing (https://www.cbd.int/abs); and (3) supporting training and capacity building in the above sectors.

Data storage and management, ensuring fast data access, data integrity and compliance with policies and regulations will also be major tasks of the proposed GRACE-RI. This infrastructure will develop solutions to connect passport, phenotypic, molecular and other types of (meta)data, both within and between studies as well as from past and future research, supporting the transformation of current germplasm collections (e.g. genebanks) into bio-digital resource centres (Mascher *et al*., 2019; Aubry, 2023). The semantic interoperability of GRACE with existing RIs will be promoted by the adoption of common ontologies, shared vocabularies that are well-established and widely used in PGR research for both data and metadata (Rocca-Serra *et al*., 2010; Selby *et al*., 2019; Papoutsoglou *et al*., 2020) as well as relevant already existing resources supporting FAIR data management (D’Anna *et al*., 2024). Moreover, enforcing permanent unique identifiers (PUIDs) to each accession (as recommended by the FAO; Alercia *et al*., 2015) and dataset (as recommended by the good practices of Open Science; Wilkinson *et al*., 2016), such as DOIs, will facilitate the tracking of resources across different repositories.

Multi-omic approaches have led to the creation of a new generation of tools designed to characterize and leverage the biodiversity of PGRs stored in collections. The implementation of these methods in GRACE-RI will disclose essential information about the accessions and make it accessible to external users. Furthermore, GRACE-RI will provide additional services to end-users: e.g. genome-wide prediction models to assess the field performance of accessions (Keilwagen *et al*., 2014; Yu *et al*., 2016; Jiang *et al*., 2021; Wang *et al*., 2024); or spatial modelling of crop performances/habitat suitability across Europe under different future climate scenarios (Wang *et al*., 2010; Booth, 2018; van Leeuwen *et al*., 2024).

## LANDSCAPE ANALYSIS OF PGR-RELATED RI IN EUROPE: GAPS AND SYNERGIES

The Landscape Analysis described in the ESFRI roadmap 2021 offers an overview of the RIs and their service portfolios accessible to European scientists and technology developers. It also highlights the scientific needs and the existing gaps in the ecosystem of the RIs, providing recommendations for future strategic investments that reduce duplication of effort and will help sustain Europe’s global leadership. The ESFRI 2021 Landscape Analysis includes RIs in Health & Food (H&F) and Environment (ENV) domains that provide support and resources to prevent biodiversity loss and promote sustainable agriculture, but there is no RI that addresses the challenges of the PGR research community. Consequently, comprehensive landscape analyses specifically focused on PGR research have not been conducted, though some reports and position papers have highlighted shortcomings in existing RIs operating in related fields of research (Morisse *et al*., 2019; Stelzl *et al*., 2023). As an example, biodiversity-oriented services have been analysed in the preparative phase of various RIs, such as the DiSSCo preparatory phase (Goodson *et al*., 2020; Smith *et al*., 2022) and ELIXIR preparatory projects, which surveyed biological databases throughout Europe (Southan and Cameron, 2009). The BiodivERsA network mapped gaps in biodiversity and ecosystem services of RIs (Manrique *et al*., 2021). Additionally, the EU-funded RISCAPE project provided an extensive overview of the major European RIs within the international research infrastructure landscape (Asmi *et al*., 2019).

As part of the PRO-GRACE project consortium and in collaboration with representatives of other European RIs, we have developed the gap and synergy analysis below. Our contributions reflect the collective perspective and strategic objectives of the proposed GRACE RI to be included in the ESFRI panorama.

The analysis involved six European RIs that cover scientific areas complementary to the conservation, management and use of PGRs, namely: ELIXIR (domain H&F – focused on biological data for life sciences), EMPHASIS (H&F – plant phenotyping), DiSSCo (ENV – digitization of natural science collections), LIFEWATCH (ENV – biodiversity and ecosystem research), METROFOOD (H&F – metrological applications for the enhancement of food quality and safety) and MIRRI (H&F – preservation, investigation and valorization of microbial resources). For comparison, recent EU-funded projects dedicated to PGRs (PRO-GRACE, HARNESSTOM, BREEDINGVALUE, AGENT, G2P-SOL, ECOBREED, INCREASE and TRADITOM – Supplementary Data Table S1) were used as a ‘proxy’ for the role and function that a future GRACE-RI could take and have. The above-mentioned RIs were compared with the future GRACE based on three different aspects: (1) research aims, as inferred from the EU-Cordis Field of Science (FS) (the CORDIS dataset –Fig. S1); (2) research products, as inferred from the Web of Science™ (WoS) categories of the papers acknowledging each RI (the WoS dataset –Figs S2 and S3); and (3) PGR-related features/services, based on a list of Key Performance Indicators (KPIs) screened and scored across the selected RIs (the KPI dataset –Table S2). A detailed description of the procedures and methods applied in the data collection can be found in Appendix S1. The three datasets obtained (CORDIS, WoS and KPIs) were subjected to minimum-spanning network analysis (Blondel *et al*., 2008), principal component analysis (PCA) and Pearson correlation analysis to find PGR-related gaps in the current European RI landscapes and potential synergies with the existing RIs.

**Fig. 1.**
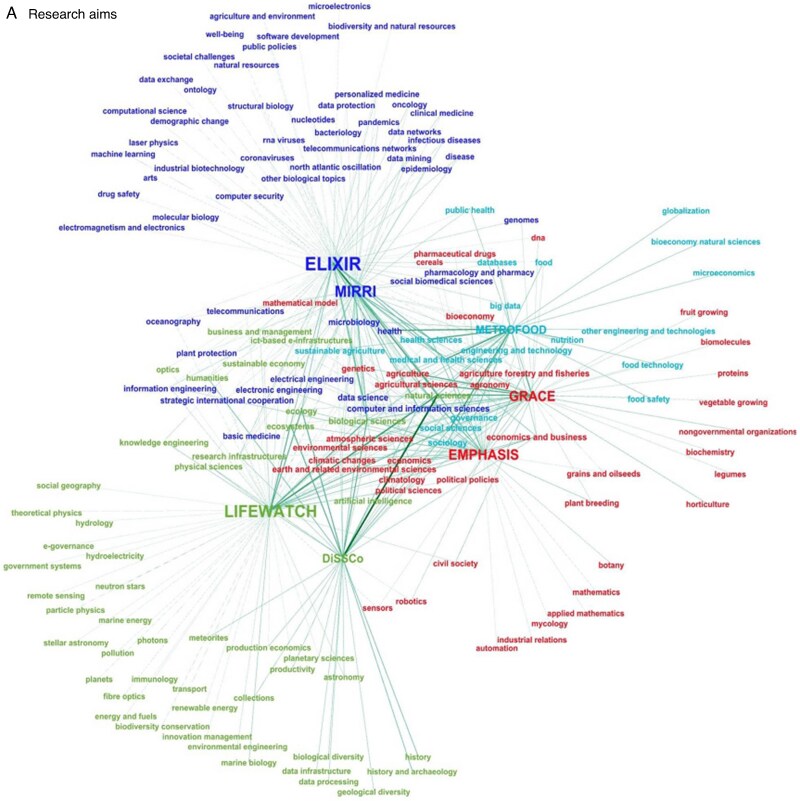

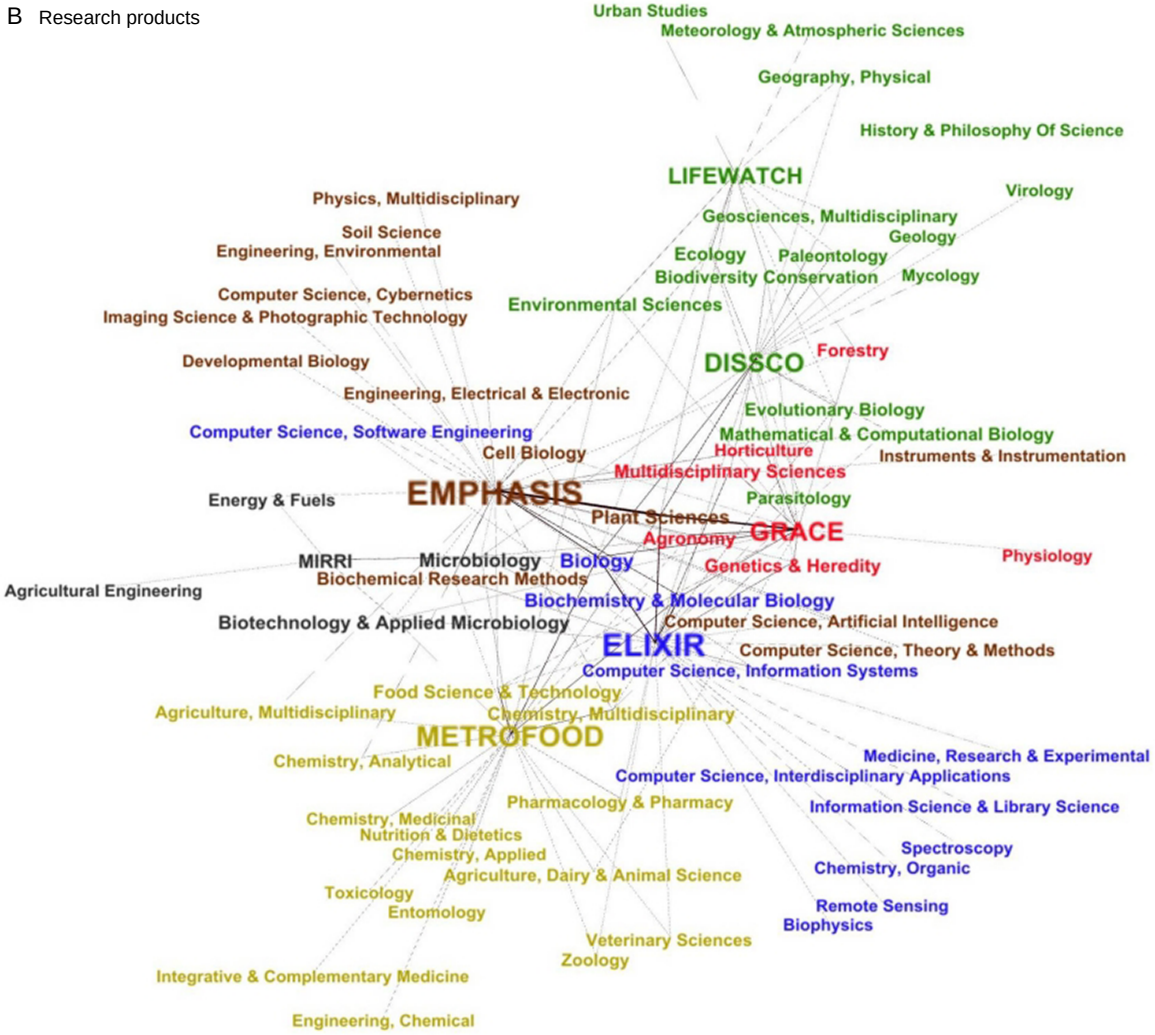

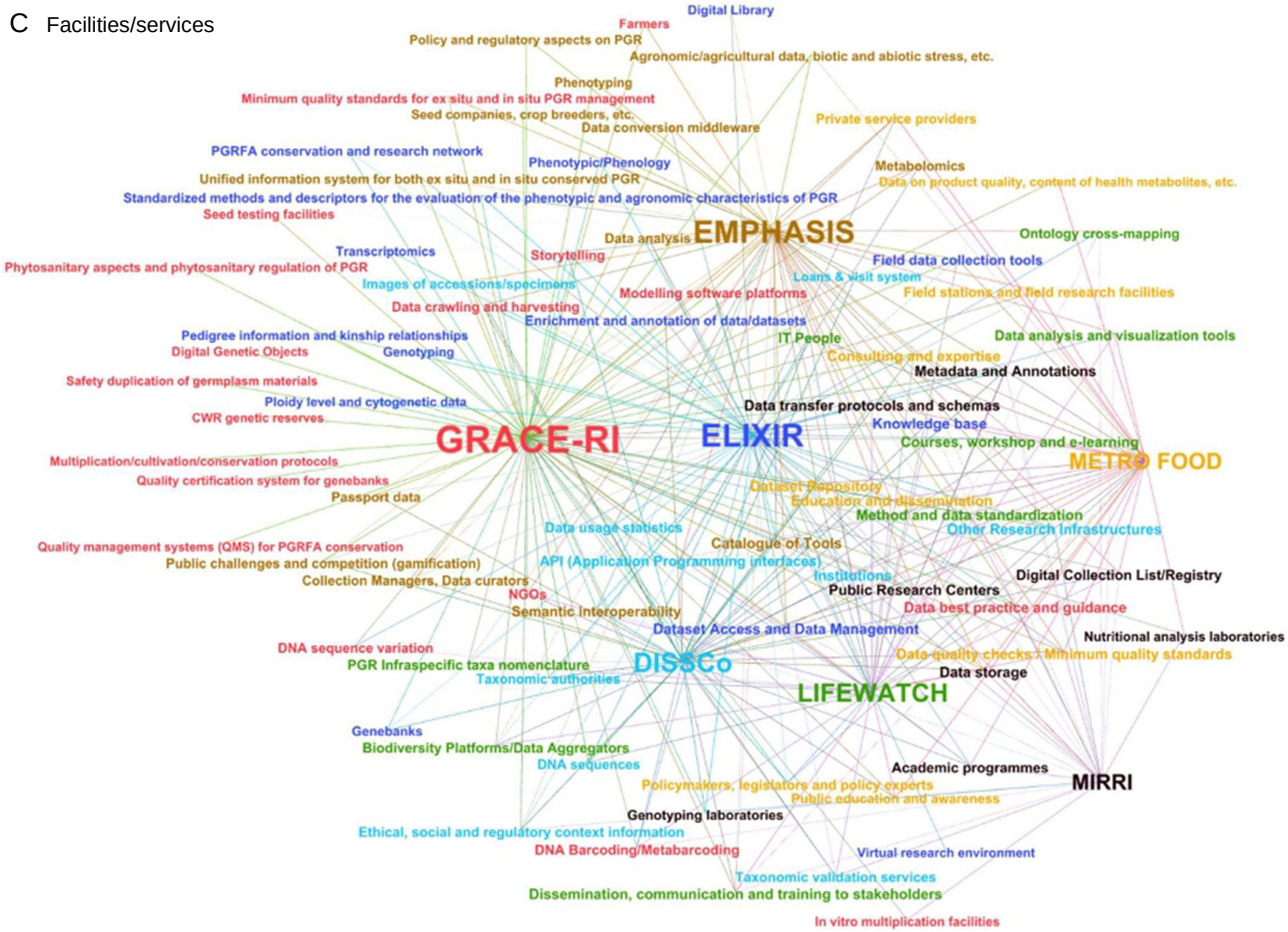
Minimum-spanning network (Force Atlas Algorithm) of the seven selected RIs based on: (A) research aims (frequency of CORDIS-EU FS of 70 projects); (B) research products (WoS categories of 314 papers); (C) service/facilities (scores of 80 KPIs). The thickness of the connecting lines is proportional to the frequency. Highly connected nodes are central in each diagram, while nodes with lower connections or specific for each RI are peripheral. The size of the RI labels is proportional to the number of interconnections with nodes. Different colours highlight different node communities after multiscalar modularity analysis. RI, research infrastructure; WoS, Web of Science™; KPI, key performance indicator. The analysis was conducted using the software Gephi version 0.10 (Bastian *et al.*, 2009).

**Fig. 2.**
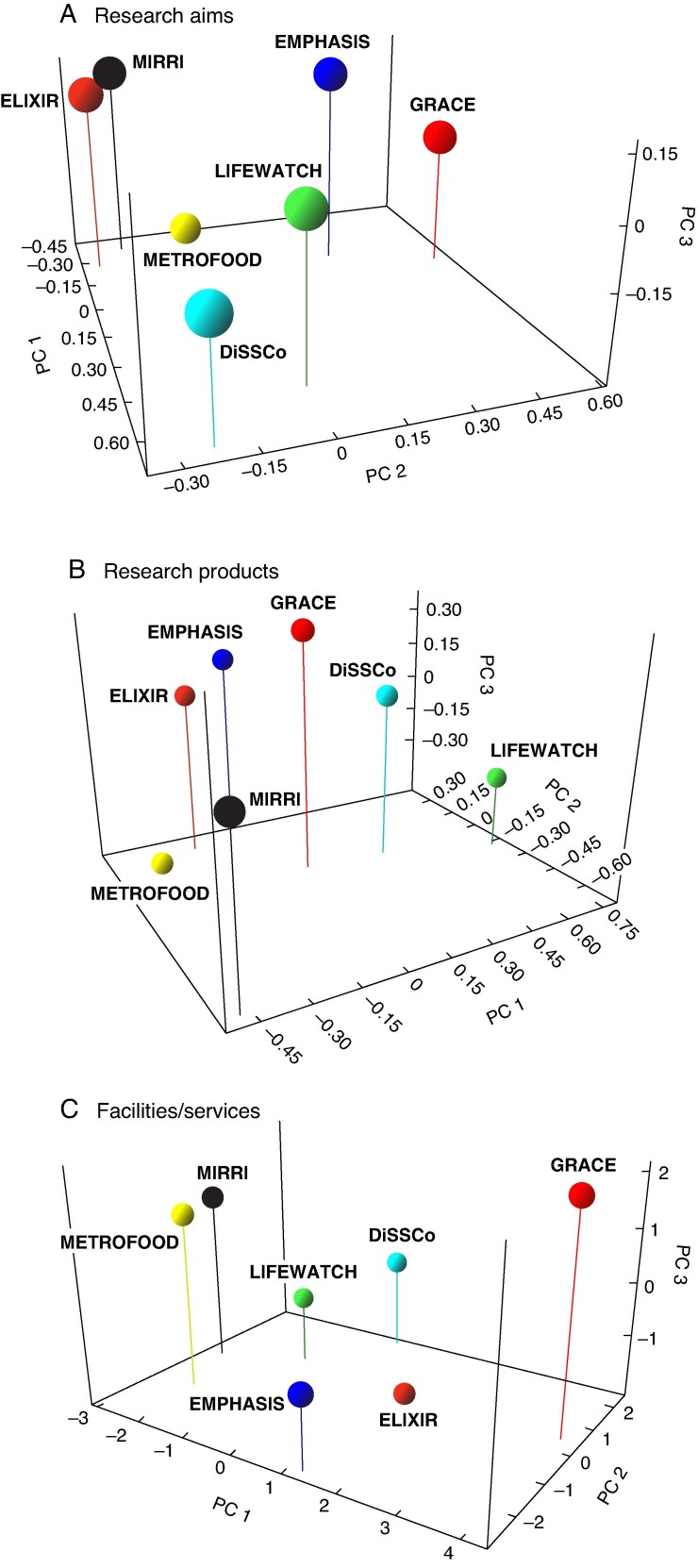
Principal component analysis (PCA) of the seven selected RIs based on: (A) research aims (frequency of CORDIS-EU FS of 70 projects); (B) research products (WoS categories of 314 papers); (C) service/facilities (scores of 80 KPIs). The first three PC axes accounted for (A) 76.23 %, (B) 80.09 % and (C) 71.27 % of the total variance. RI, research infrastructure; WoS, Web of Science™; KPI, key performance indicator. The analysis was conducted using the software Past 4.02 (Hammer *et al.*, 2001).

Minimum-spanning network analysis of research aims and products revealed a highly interconnected and correlated landscape in the EU research infrastructure ecosystem (Fig. 1A, B), with ELIXIR, MIRRI and LIFEWATCH being the most interconnected RIs in terms of research aims, and EMPHASIS, ELIXIR and METROFOOD RIs in terms of research products. In both cases, GRACE-RI (as represented by recent EU-funded research projects on PGRs) was half-ranking for the number of connections, with minimal overlaps with other RIs. However, it is worth underlining that the different maturity levels of the selected RIs may affect their interconnectivity, e.g. a higher number of publications could have been indexed on the WoS platform for the ‘older’ RIs (Supplementary Data Appendix S1). Nonetheless, the above analyses revealed a distinct grouping of the GRACE ‘proxy’ compared with the selected RIs, showing close relationships only with EMPHASIS regarding research aims (Fig. 1A). The results of PCA in terms of research aims confirmed the relationship between EMPHASIS and GRACE-RI and showed a correlation between DiSSCo and LIFEWATCH on the one hand and ELIXIR, MIRRI and METROFOOD on the other (Fig. 2A). The analysis based on research products identified two distinct groups: (1) DiSSCo and LIFEWATCH, and (2) ELIXIR, EMPHASIS and METROFOOD. MIRRI was classified as an outlier probably due to the low number of related papers found. In this context, the positioning of the future GRACE seemed to be mid-way among the other RIs (Fig. 2B). The analysis of PGR-related features and services based on the KPI dataset highlighted the distinctiveness of GRACE among the selected RIs (Figs 1C and 2C) and its potential to develop services that are currently missing in the landscape of EU research infrastructures. GRACE-RI was identified as having unique KPIs related to PGRs, including ‘quality management system for PGR/genebanks’, ‘safety duplications of PGR materials’, ‘multiplication/cultivation/conservation protocols’, and ‘phytosanitary aspects and regulation’ (Table S2). Furthermore, the results of both network analysis and PCA suggested a set of initiatives that GRACE will undertake (see below) to integrate, enhance and support cutting-edge PGR research in Europe.

**Fig. 3.**
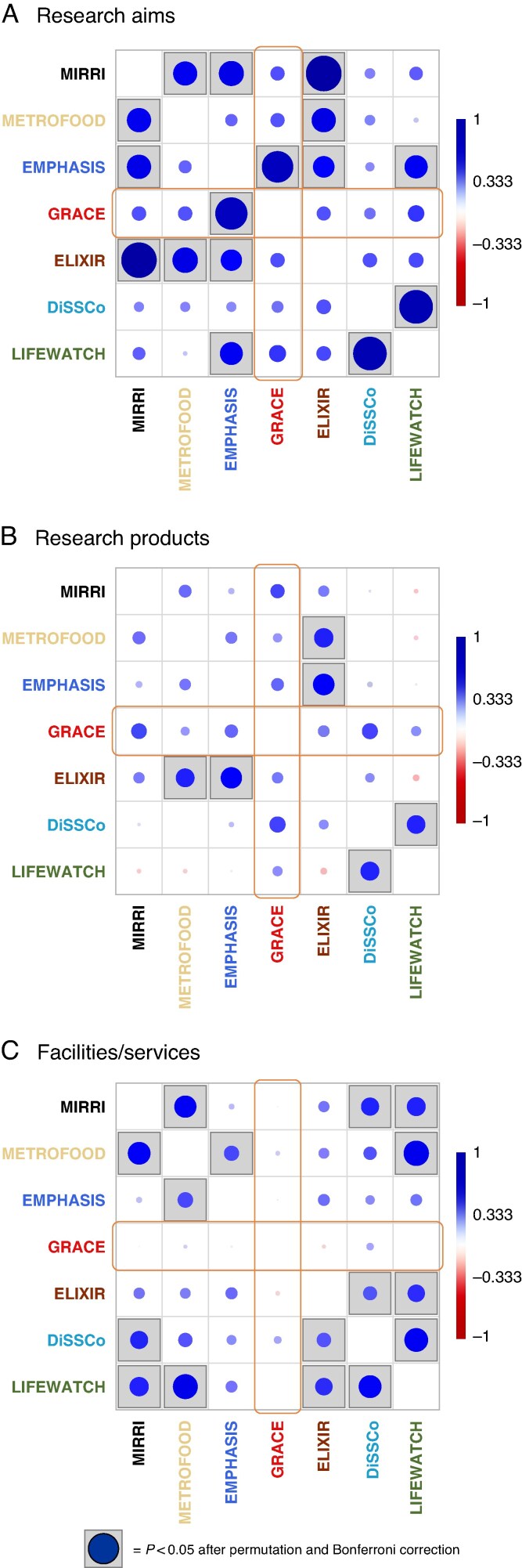
Pairwise Pearson’s correlations between the seven RIs analysed based on: (A) research aims (frequency of CORDIS-EU FS of 70 projects); (B) research products (WoS categories of 314 papers); and (C) features/services (scores of 80 KPIs). Boxed circles indicate significant pairwise correlations (*P* < 0.05) after permutation and Bonferroni’s correction. RI, research infrastructure; WoS, Web of Science™; KPI, key performance indicator. The analysis was conducted using the software Past 4.02 (Hammer *et al.*, 2001).

The Pearson correlation coefficients calculated between the seven RIs using the three datasets showed many significant correlations (Fig. 3). For research aims, EMPHASIS had the largest number of significant correlations with other RIs, including with GRACE (Fig. 3A). Conversely, no significant correlations were observed between GRACE and other RIs surveyed for both research products and facilities/services (Fig. 3B, C). Overall, the results of the above analysis proved that GRACE has the potential to bridge gaps in the existing RIs by implementing current PGR research in Europe and interconnecting DiSSCo and LIFEWATCH on one side with ELIXIR, EMPHASIS and METROFOOD on the other.

The potential link between GRACE, ELIXIR and METROFOOD will further implement the use of dedicated EU facilities and resources to achieve the transcriptomic, metabolomic and proteomic characterization of biological tissues from plants having (or not) food and nutritional interest, thus complementing genomic information on these resources. On the other hand, the possible link between GRACE and EMPHASIS will further boost the use of facilities, resources and services for plant phenotyping across Europe. The inclusion of GRACE-RI into the ESFRI landscape will broaden the network of stakeholders for both platforms, ultimately maximizing the results of the investments made.

In summary, the quantitative approach used in this gap and synergy analysis highlighted the distinctiveness (in terms of features/services) of the future GRACE dedicated to PGRs, compared with the selected RIs, and its complementarity (in terms of research aims and research products) in the current PGR-related landscape of research infrastructures in Europe. In other words, GRACE-RI will be ‘central’ by closing the gaps in PGR research through multidisciplinary science, but ‘outstanding’ for the services provided to end users.

## CONCLUSIONS

To streamline and strengthen PGR conservation and use in wider, coherent policies and programmes and to raise awareness of the evolutionary potential of PGRs, additional collaborative actions are needed at national and European levels. Key collaboration areas include (1) *in situ* and *ex situ* PGR conservation; (2) on-farm PGR management; (3) promoting PGR use in research and breeding; (4) PGR documentation and information enhancements; and (5) scientific and technical exchange and networking.

In this context, the establishment of GRACE-RI is crucial for addressing the challenges faced by the European PGR ecosystem, promoting a common effort across existing ESFRI RIs, facilitating a seamless exchange of information among them and making access to PGR resources easier for end-users. The deployment of the proposed RI could accelerate progress toward sustainable and resilient agriculture and provide an unparalleled contribution to global food and nutrition security as well as plant biodiversity conservation.

The gap analysis showed that the proposed GRACE-RI could address the current gaps in the PGR-related landscape of EU RIs by fostering collaborations and synergies between existing RIs in complementary fields. On the other hand, failure to implement GRACE-RI would deepen the current inefficiency in the European PGR collections, especially considering the new challenges related to climate, environment and food security. It would also fail to create those synergistic effects unfolding from the interaction with the complementary RIs, thus reducing their potential impact on European well-being.

As highlighted by the landscape analysis included in the ESFRI Roadmap 2021, the research infrastructures of the H&F domain represent a pivotal cross-sectorial interaction platform interconnecting the environmental domains, sustainability of food production in the context of climate change and human health. In our perspective, the novel GRACE-RI dedicated to PGRs represents the missing link along the chain connecting health, food and environment, and will further expand interactions within and across existing RIs through an interdisciplinary approach to cross-cutting PGR research.

## Supplementary Material

mcaf092_Supplementary_Data

## SUPPLEMENTARY DATA

Supplementary data are available at *Annals of Botany* online and consist of the following. Table S1: The complete list of the 70 preparatory/collaborative projects analysed in this study and their parent RIs. Table S2: List of the 80 key performance indicators (KPIs) selected to evaluate the activities of the selected RIs supporting PGR research in Europe. Figure S1: Partition of the preparatory/collaborative RI projects by disciplines derived from the CORDIS-EC Fields of Science (FS). Figure S2: Partition of PGR-related papers retrieved from the Clarivate Web of Science™ platform using the name/acronym (outer circle) of the preparatory/collaborative projects considered in this analysis, and their parent RI (inner circle). Figure S3: Partition of papers (*N* = 314) by disciplines derived from the Web of Science categories.

## References

[mcaf092-B1] Antonelli A, Fry C, Smith RJ, et al *State of the World’s Plants and Fungi 2023. Tackling the Nature Emergency: Evidence, gaps and priorities*. Royal Botanic Gardens, Kew. 2023. doi:10.34885/wnwn-6s63.

[mcaf092-B2] Asmi A, Ryan L, Salmon E, et al *International Research Infrastructure Landscape 2019*. EC H2020 programme. 2019. doi:10.5281/ZENODO.3539254. Version 1

[mcaf092-B3] Goodson H, Gödderz K, Casino A, Koureas D, Walton S. *Positioning DiSSCo among other research infrastructures*. ICEDIG Project. 2020. doi: 10.5281/zenodo.3733032.

[mcaf092-B4] Southan C, Cameron G. D2.1: Database Provider Survey report for ELIXIR Work Package 2. Zenodo. 2009. doi:10.5281/zenodo.576013.

[mcaf092-B5] Agovino M, Casaccia M, Ciommi M, Ferrara M, Marchesano K. 2019. Agriculture, climate change and sustainability: the case of EU-28. Ecological Indicators 105: 525–543. doi:10.1016/j.ecolind.2018.04.064

[mcaf092-B6] Alercia A, Diulgheroff S, Mackay M. **2015.** *FAO/Bioversity Multi-Crop Passport Descriptors V.2.1 [MCPD V.2.1]*. https://alliancebioversityciat.org/publications-data/faobioversity-multi-crop-passport-descriptors-v21-mcpd-v21-december-2015 (accessed 29 April 2025).

[mcaf092-B7] Aubry S . 2023. Genebanking plant genetic resources in the postgenomic era. Agriculture and Human Values 40: 961–971. doi:10.1007/s10460-023-10417-7

[mcaf092-B8] Bajocco S, Smiraglia D, Scaglione M, Raparelli E, Salvati L. 2018. Exploring the role of land degradation on agricultural land use change dynamics. Science of The Total Environment 636: 1373–1381. doi:10.1016/j.scitotenv.2018.04.412 29913598

[mcaf092-B9] Barchi L, Aprea G, Rabanus-Wallace MT, et al 2023. Analysis of >3400 worldwide eggplant accessions reveals two independent domestication events and multiple migration-diversification routes. The Plant Journal 116: 1667–1680. doi:10.1111/tpj.16455 37682777

[mcaf092-B10] Barchi L, Rabanus-Wallace MT, Prohens J, et al 2021. Improved genome assembly and pan-genome provide key insights into eggplant domestication and breeding. The Plant Journal 107: 579–596. doi:10.1111/tpj.15313 33964091 PMC8453987

[mcaf092-B11] Bastian M, Heymann S, Jacomy M. 2009. Gephi: an open source software for exploring and manipulating networks. Proceedings of the International AAAI Conference on Web and Social Media 3: 361–362. doi:10.1609/icwsm.v3i1.13937

[mcaf092-B12] Bhadouria R, Singh R, Singh VK, et al 2019. Agriculture in the era of climate change: consequences and effects. In: Choudhary KK, Kumar A, Singh AK. eds. Climate change and agricultural ecosystems. Sawston: Woodhead Publishing, 1–23.

[mcaf092-B13] Blätke M-A, Szymanski JJ, Gladilin E, Scholz U, Beier S. 2021. Editorial: advances in applied bioinformatics in crops. Frontiers in Plant Science 12: 640394. DOI: 10.3389/fpls.2021.640394 33679855 PMC7928291

[mcaf092-B14] Blondel VD, Guillaume J-L, Lambiotte R, Lefebvre E. 2008. Fast unfolding of communities in large networks. Journal of Statistical Mechanics: Theory and Experiment 2008: P10008. doi:10.1088/1742-5468/2008/10/P10008

[mcaf092-B15] Booth TH . 2018. Species distribution modelling tools and databases to assist managing forests under climate change. Forest Ecology and Management 430: 196–203. doi:10.1016/j.foreco.2018.08.019

[mcaf092-B16] Brink M, van Hintum T. 2020. Genebank operation in the arena of access and benefit-sharing policies. Frontiers in Plant Science 10: 1712. doi:10.3389/fpls.2019.01712 32038684 PMC6987393

[mcaf092-B17] Calle García J, Guadagno A, Paytuvi-Gallart A, et al 2022. PRGdb 4.0: an updated database dedicated to genes involved in plant disease resistance process. Nucleic Acids Research 50: D1483–D1490. doi:10.1093/nar/gkab1087 34850118 PMC8729912

[mcaf092-B18] Cheng S, Feng C, Wingen LU, et al 2024. Harnessing landrace diversity empowers wheat breeding. Nature 632: 823–831. doi:10.1038/s41586-024-07682-9 38885696 PMC11338829

[mcaf092-B19] D’Anna F, Jareborg N, Jetten M, et al 2024. A research data management (RDM) community for ELIXIR. F1000Research 13: ELIXIR-230. doi:10.12688/f1000research.146301.2 PMC11474151 39410979

[mcaf092-B20] Darbyshire I, Anderson S, Asatryan A, et al 2017. Important plant areas: revised selection criteria for a global approach to plant conservation. Biodiversity and Conservation 26: 1767–1800. doi:10.1007/s10531-017-1336-6

[mcaf092-B21] ECPGR . 2021. *Plant Genetic Resources Strategy for Europe*. Rome, Italy: European Cooperative Programme for Plant Genetic Resources. https://www.ecpgr.org/resources/ecpgr-publications/publication/plant-genetic-resources-strategy-for-europe-2021 (accessed 29 Aprril 2025).

[mcaf092-B22] El Hanafi S, Jiang Y, Kehel Z, et al 2023. Genomic predictions to leverage phenotypic data across genebanks. Frontiers in Plant Science 14: 1227656. doi:10.3389/fpls.2023.1227656 37701801 PMC10493331

[mcaf092-B23] Fradgley NS, Bacon J, Bentley AR, et al 2023. Prediction of near-term climate change impacts on UK wheat quality and the potential for adaptation through plant breeding. Global Change Biology 29: 1296–1313. doi:10.1111/gcb.16552 36482280 PMC10108302

[mcaf092-B24] Gabdank I, Chan ET, Davidson JM, et al 2018. Prevention of data duplication for high throughput sequencing repositories. Database 2018: bay008. doi:10.1093/database/bay008 29688363 PMC5829560

[mcaf092-B25] Gonzalo MJ, Nájera I, Baixauli C, et al 2021. Identification of tomato accessions as source of new genes for improving heat tolerance: from controlled experiments to field. BMC Plant Biology 21: 345. doi:10.1186/s12870-021-03104-4 34294034 PMC8296629

[mcaf092-B26] Goritschnig S, Weise S, Guzzon F, et al 2025. Strengthening European research cooperation on plant genetic resources conservation and use. Genetic Resources 119–134. doi:10.46265/genresj.LUZJ7324

[mcaf092-B27] Gupta M, Salgotra RK, Chauhan BS. 2020. Next-generation sequencing technologies and their implications for efficient utilization of genetic resources. In: Salgotra RK, Zargar SM. eds. Rediscovery of genetic and genomic resources for future food security, Singapore: Springer, 239–250.

[mcaf092-B29] Halewood M, Chiurugwi T, Sackville Hamilton R, et al 2018. Plant genetic resources for food and agriculture: opportunities and challenges emerging from the science and information technology revolution. The New Phytologist 217: 1407–1419. doi:10.1111/nph.14993 29359808

[mcaf092-B30] Hammer Ø, Harper DAT, Ryan PD. 2001. PAST: paleontological statistics software package for education and data analysis. Palaeontologia Electronica 4: 9.

[mcaf092-B31] Hannah L, Ikegami M, Hole DG, et al 2013. Global climate change adaptation priorities for biodiversity and food security. PLoS One 8: e72590. doi:10.1371/journal.pone.0072590 23991125 PMC3749124

[mcaf092-B32] Jägermeyr J, Müller C, Ruane AC, et al 2021. Climate impacts on global agriculture emerge earlier in new generation of climate and crop models. Nature Food 2: 873–885. doi:10.1038/s43016-021-00400-y 37117503

[mcaf092-B33] Jamil IN, Remali J, Azizan KA, et al 2020. Systematic multi-omics integration (MOI) approach in plant systems biology. Frontiers in Plant Science 11: 944. doi:10.3389/fpls.2020.00944 32754171 PMC7371031

[mcaf092-B34] Jia J, Li H, Zhang X, Li Z, Qiu L. 2017. Genomics-based plant germplasm research (GPGR). The Crop Journal 5: 166–174. doi:10.1016/j.cj.2016.10.006

[mcaf092-B35] Jiang Y, Weise S, Graner A, Reif JC. 2021. Using genome-wide predictions to assess the phenotypic variation of a Barley (*Hordeum* sp.) gene bank collection for important agronomic traits and passport information. Frontiers in Plant Science 11: 604781. doi:10.3389/fpls.2020.604781 33505414 PMC7829250

[mcaf092-B36] Keilwagen J, Kilian B, Özkan H, et al 2014. Separating the wheat from the chaff—a strategy to utilize plant genetic resources from ex situ genebanks. Scientific Reports 4: 5231. doi:10.1038/srep05231 24912875 PMC4050481

[mcaf092-B37] Khoury CK, Brush S, Costich DE, et al 2022. Crop genetic erosion: understanding and responding to loss of crop diversity. New Phytologist 233: 84–118. doi:10.1111/nph.17733 34515358

[mcaf092-B38] König P, Beier S, Basterrechea M, et al 2020. BRIDGE—a visual analytics web tool for barley genebank genomics. Frontiers in Plant Science 11: 701. doi:10.3389/fpls.2020.00701 32595658 PMC7300248

[mcaf092-B39] Kotni P, van Hintum T, Maggioni L, Oppermann M, Weise S. 2023. EURISCO update 2023: the European search catalogue for plant genetic resources, a pillar for documentation of genebank material. Nucleic Acids Research 51: D1465–D1469. doi:10.1093/nar/gkac852 36189883 PMC9825528

[mcaf092-B40] Malhi GS, Kaur M, Kaushik P. 2021. Impact of climate change on agriculture and its mitigation strategies: a review. Sustainability 13: 1318. doi:10.3390/su13031318

[mcaf092-B41] Manrique E, Blery C, Le Roux X, Mandon C; all BiodivERsA partners. 2021. *BiodivERsA Mapping of Biodiversity Research Infrastructures. BiodivERsa Report*. https://www.biodiversa.eu/wp-content/uploads/2022/12/mapping-biodiversity-research-infrastructures.pdf (accessed 29 April 2025).

[mcaf092-B42] Mascher M, Schreiber M, Scholz U, Graner A, Reif JC, Stein N. 2019. Genebank genomics bridges the gap between the conservation of crop diversity and plant breeding. Nature Genetics 51: 1076–1081. doi:10.1038/s41588-019-0443-6 31253974

[mcaf092-B43] Maxted N, Kell S. **2021.** *European network for in situ conservation and sustainable use of plant genetic resources*. Birmingham, UK: University of Birmingham. https://more.bham.ac.uk/farmerspride/wp-content/uploads/sites/19/2021/11/D4.4_European_in_situ_PGR_conservation_network.pdf. (accessed 29 April 2025).

[mcaf092-B44] McCouch S, Navabi ZK, Abberton M, et al 2020. Mobilizing crop biodiversity. Molecular Plant 13: 1341–1344. doi:10.1016/j.molp.2020.08.011 32835887

[mcaf092-B45] Mellidou I, Koukounaras A, Frusciante S, et al 2023. A metabolome and transcriptome survey to tap the dynamics of fruit prolonged shelf-life and improved quality within Greek tomato germplasm. Frontiers in Plant Science 14: 1267340. doi:10.3389/fpls.2023.1267340 37818313 PMC10560995

[mcaf092-B46] Morisse M, Wells D, Alary P-E, et al **2019.** *Gap Analysis*. CORDIS EC. https://ec.europa.eu/research/participants/documents/downloadPublic?documentIds=080166e5cb9e5662&appId=PPGMS (accessed 29 April 2025).

[mcaf092-B47] OHEJP . **2023.** *Strategic Research and Innovation Agenda*. https://onehealthejp.eu/wp-content/uploads/2023/05/OHEJP-Strategic-Research-and-Innovation-Agenda_05.05.23.pdf (accessed 29 April 2025).

[mcaf092-B48] Papoutsoglou EA, Faria D, Arend D, et al 2020. Enabling reusability of plant phenomic datasets with MIAPPE 1.1. The New Phytologist 227: 260–273. doi:10.1111/nph.16544 32171029 PMC7317793

[mcaf092-B49] Pilling D, Bélanger J, Diulgheroff S, et al 2020. Global status of genetic resources for food and agriculture: challenges and research needs: global status of genetic resources for food and agriculture. Genetic Resources 1: 4–16. doi:10.46265/genresj.2020.1.4-16

[mcaf092-B50] Rocca-Serra P, Brandizi M, Maguire E, et al 2010. ISA software suite: supporting standards-compliant experimental annotation and enabling curation at the community level. Bioinformatics (Oxford, England) 26: 2354–2356. doi:10.1093/bioinformatics/btq415 20679334 PMC2935443

[mcaf092-B51] Sanchez D, Sadoun SB, Mary-Huard T, Allier A, Moreau L, Charcosset A. 2023. Improving the use of plant genetic resources to sustain breeding programs’ efficiency. Proceedings of the National Academy of Sciences of the United States of America 120: e2205780119. doi:10.1073/pnas.2205780119 36972431 PMC10083577

[mcaf092-B52] Santos JA, Fraga H, Malheiro AC, et al 2020. A review of the potential climate change impacts and adaptation options for European viticulture. Applied Sciences 10: 3092. doi:10.3390/app10093092

[mcaf092-B53] Selby P, Abbeloos R, Backlund JE, et al 2019. BrAPI-an application programming interface for plant breeding applications. Bioinformatics (Oxford, England) 35: 4147–4155. doi:10.1093/bioinformatics/btz190 30903186 PMC6792114

[mcaf092-B54] Silva G. **2018.** *Feeding the world in 2050 and beyond—Part 1: Productivity challenges*. https://www.canr.msu.edu/news/feeding-the-world-in-2050-and-beyond-part-1. (accessed 29 April 2025).

[mcaf092-B55] Singh N, Wu S, Raupp WJ, et al 2019. Efficient curation of genebanks using next generation sequencing reveals substantial duplication of germplasm accessions. Scientific Reports 9: 650. doi:10.1038/s41598-018-37269-0 30679756 PMC6346010

[mcaf092-B56] Skendžić S, Zovko M, Živković IP, Lešić V, Lemić D. 2021. The impact of climate change on agricultural insect pests. Insects 12: 440. doi:10.3390/insects12050440 34066138 PMC8150874

[mcaf092-B57] Smith VS, French L, Vincent S, et al 2022. Research infrastructure contact zones: a framework and dataset to characterise the activities of major biodiversity informatics initiatives. Biodiversity Data Journal 10: e82953. doi:10.3897/BDJ.10.e82953 36761622 PMC9848541

[mcaf092-B58] Smith S, Nickson TE, Challender M. 2021. Germplasm exchange is critical to conservation of biodiversity and global food security. Agronomy Journal 113: 2969–2979. doi:10.1002/agj2.20761

[mcaf092-B59] Stelzl T, Tsimidou MZ, Belc N, Zoani C, Rychlik M. 2023. Building a novel strategic research agenda for METROFOOD-RI: design process and multi-stakeholder engagement towards thematic prioritization. Frontiers in Nutrition 10: 1151611. doi:10.3389/fnut.2023.1151611 37426195 PMC10327297

[mcaf092-B60] Swarup S, Cargill EJ, Crosby K, Flagel L, Kniskern J, Glenn KC. 2021. Genetic diversity is indispensable for plant breeding to improve crops. Crop Science 61: 839–852. doi:10.1002/csc2.20377

[mcaf092-B61] Thuiller W, Lavergne S, Roquet C, Boulangeat I, Lafourcade B, Araujo MB. 2011. Consequences of climate change on the tree of life in Europe. Nature 470: 531–534. doi:10.1038/nature09705 21326204

[mcaf092-B62] Thuiller W, Lavorel S, Araújo MB, Sykes MT, Prentice IC. 2005. Climate change threats to plant diversity in Europe. Proceedings of the National Academy of Sciences of the United States of America 102: 8245–8250. doi:10.1073/pnas.0409902102 15919825 PMC1140480

[mcaf092-B63] Tripodi P, Rabanus-Wallace MT, Barchi L, et al 2021. Global range expansion history of pepper (Capsicum spp.) revealed by over 10,000 genebank accessions. Proceedings of the National Academy of Sciences of the United States of America 118: e2104315118. doi:10.1073/pnas.2104315118 34400501 PMC8403938

[mcaf092-B64] van Hintum T, Wijker E. 2024. Quality management in a genebank environment: principles and experiences at the centre for genetic resources, The Netherlands (CGN). Genetic Resources 6–12. doi:10.46265/genresj.RFXB357039776567

[mcaf092-B65] van Leeuwen C, Sgubin G, Bois B, et al 2024. Climate change impacts and adaptations of wine production. Nature Reviews Earth & Environment 5: 258–275. doi:10.1038/s43017-024-00521-5

[mcaf092-B66] van Treuren R, van Hintum TJL. 2014. Next-generation genebanking: plant genetic resources management and utilization in the sequencing era. Plant Genetic Resources 12: 298–307. doi:10.1017/S1479262114000082

[mcaf092-B67] Volk GM, Byrne PF, Coyne CJ, Flint-Garcia S, Reeves PA, Richards C. 2021. Integrating genomic and phenomic approaches to support plant genetic resources conservation and use. Plants 10: 2260. doi:10.3390/plants10112260 34834625 PMC8619436

[mcaf092-B68] von Steimker J, Tripodi P, Wendenburg R, et al 2024. The genetic architecture of the pepper metabolome and the biosynthesis of its signature capsianoside metabolites. Current Biology 34: 4209–4223.e3. doi:10.1016/j.cub.2024.07.098 39197460

[mcaf092-B69] Wambugu PW, Ndjiondjop M-N, Henry RJ. 2018. Role of genomics in promoting the utilization of plant genetic resources in genebanks. Briefings in Functional Genomics 17: 198–206. doi:10.1093/bfgp/ely014 29688255 PMC5967547

[mcaf092-B70] Wang P, Lehti-Shiu MD, Lotreck S, Segura Abá K, Krysan PJ, Shiu S-H. 2024. Prediction of plant complex traits via integration of multi-omics data. Nature Communications 15: 6856. doi:10.1038/s41467-024-50701-6 PMC11316822 39127735

[mcaf092-B71] Wang T, O’Neill GA, Aitken SN. 2010. Integrating environmental and genetic effects to predict responses of tree populations to climate. Ecological Applications 20: 153–163. doi:10.1890/08-2257.1 20349837

[mcaf092-B72] Weise S, Lohwasser U, Oppermann M. 2020. Document or lose it—on the importance of information management for genetic resources conservation in genebanks. Plants 9: 1050. doi:10.3390/plants9081050 32824806 PMC7465628

[mcaf092-B73] Welcker C, Spencer NA, Turc O, et al 2022. Physiological adaptive traits are a potential allele reservoir for maize genetic progress under challenging conditions. Nature Communications 13: 3225. doi:10.1038/s41467-022-30872-w PMC9184527 35680899

[mcaf092-B74] Wilkinson MD, Dumontier M, Aalbersberg IJJ, et al 2016. The FAIR guiding principles for scientific data management and stewardship. Scientific Data 3: 160018. doi:10.1038/sdata.2016.18 26978244 PMC4792175

[mcaf092-B75] Yu X, Li X, Guo T, et al 2016. Genomic prediction contributing to a promising global strategy to turbocharge gene banks. Nature Plants 2: 16150. doi:10.1038/nplants.2016.150 27694945

