# Supplementary material for: A gap and synergy analysis of the European research infrastructure (RI) ecosystem: advancing the novel GRACE-RI dedicated to plant genetic resources
Source: Ann Bot. 2025 Jun 10;136(2):275–85. doi: 10.1093/aob/mcaf092 (PMC12445849; doi:10.1093/aob/mcaf092)
Supplement: mcaf092_Supplementary_Data [file mcaf092_supplementary_data.zip › aob-25033-s03.docx]

### **APPENDIX 1 – DATASET PREPARATION**

1.1 - Analysis of the research aims

A list of preparatory, collaborative, and joint projects involving each selected RIs was compiled using information gathered from their websites. The analysis focused on the overall activities of the selected RIs, including, but not limited to, PGR research. Each project in the list was searched on the CORDIS-EC platform (<https://cordis.europa.eu/>), and the associated Fields of Science (FS) were counted and assigned to the corresponding (parent) RI. If two RIs participated in the same project, the project and their associated FS were attributed to both parent RIs.

A total of 70 EU-funded projects were selected (see **Tab. S1**) and categorized by FS, reflecting their research aims and those of their parent RIs. Overall, 156 distinct FS were found, which were grouped into 39 broader disciplines to minimize the heteroscedasticity of the matrix (**Fig. S1**), which was made up of 7 columns (6 parent RIs + the GRACE-RI proxy) and 39 rows (the disciplines derived from the 156 FS). The frequency of the disciplines in each parent RI was converted in percentage (x) and then transformed as asin(sqrt(x)) to avoid departures from normality.

1.2 - Analysis of the research products

The selected RIs were also compared based on their support of scientific products in PGR research, i.e., PGR-related papers indexed in the Clarivate Web of Science™ (WoS) platform (<https://www.webofscience.com/>) that explicitly acknowledged one of the 70 mentioned projects or its parent RI in the funding information or acknowledgments. We searched only for experimental works published in journals endowed with Impact Factor, while position papers, commentaries, perspectives, etc. were intentionally discarded. As for GRACE-RI, only papers acknowledging the afore-mentioned EU-funded projects on PGR were included. The literature search was carried out in August-September 2023.

To ensure a thorough retrieval of information, the search included the full names or acronyms of each RI and their associated projects. However, due to a high number of unrelated results (e.g., 775 records for "ELIXIR"), additional filters like "plant*" and "crop*" were applied. The retrieved papers were accurately scrutinized to exclude those unrelated to PGR. This process resulted in a final dataset of 314 articles covering all selected RIs, though the number of papers varied significantly across RIs: 98 for EMPHASIS, 70 for ELIXIR, 48 for GRACE, 42 for METROFOOD, 40 for DiSSCo, 14 for LIFEWATCH, and only 3 for MIRRI.

Each paper retrieved was marked by one or more WoS categories, which reflected the research area, or the fields of science investigated. Overall, the total number of unique WoS categories in the dataset was 63. To reduce the scattering of data across categories and the heteroscedasticity of the dataset, the 63 distinct WoS categories detected were grouped in 31 broader disciplines, which reflected the research areas supported by the RIs included in the analysis (**Fig. S3**). The frequency of papers marked by each discipline was recorded for each parent RI, obtaining a matrix of 7 RIs × 31 disciplines. Again, the frequency of the disciplines recorded for each parent RI was converted in percentage (x), i.e., the percentage of papers marked by a given category out of the total number of papers attributed to the parent RI, and then transformed as asin(sqrt(x)).

1.3 - Analysis of PGR-related features/services

To identify potential overlaps and gaps between the novel GRACE-RI and the existing European RIs, a set of Key Performance Indicators (KPIs) specific to PGR-related features/services was developed (see **Tab. S2**). Representatives from each selected RI were asked to score these KPIs as present (1) or absent (0). Some experts provided intermediate scores (0.1-0.9) for partially present features. This process resulted in a matrix of 7 RIs scored for 80 KPIs, which was submitted to statistical analysis to identify common and unique features.
