# Supplementary material for: A gap and synergy analysis of the European research infrastructure (RI) ecosystem: advancing the novel GRACE-RI dedicated to plant genetic resources
Source: Ann Bot. 2025 Jun 10;136(2):275–85. doi: 10.1093/aob/mcaf092 (PMC12445849; doi:10.1093/aob/mcaf092)
Supplement: mcaf092_Supplementary_Data [file mcaf092_supplementary_data.zip › aob-25033-s05.docx]

### SUPPLEMENTARY FIGURES


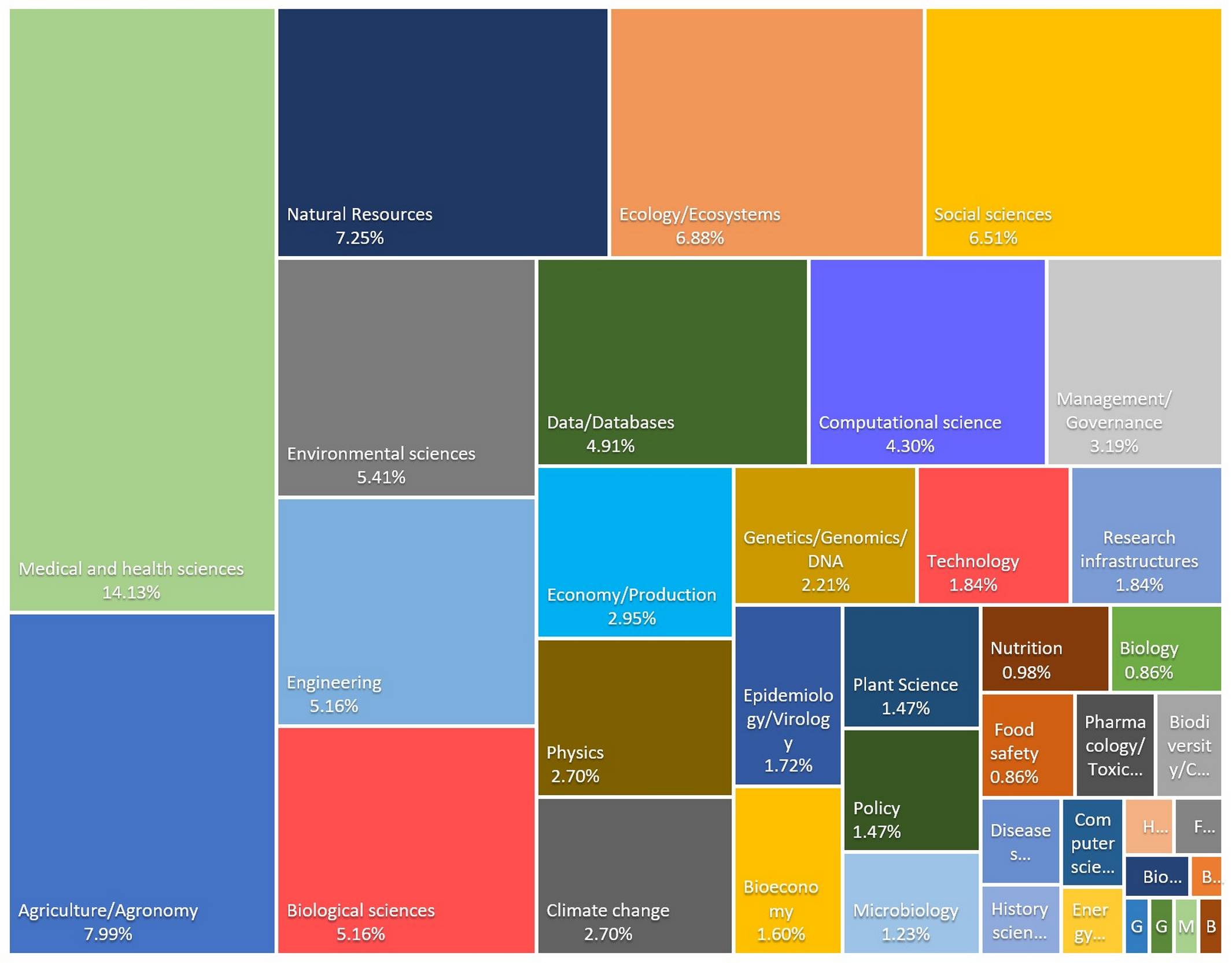


FIG. S1 - Partition of the preparatory/collaborative RI projects by disciplines derived from the CORDIS-EC Fields of Science (FS). To reduce the scattering of data across FS and the heteroscedasticity of the dataset, the 156 distinct FS detected were grouped in 39 large disciplines, which reflected the research aims of the 70 projects included in the analysis.


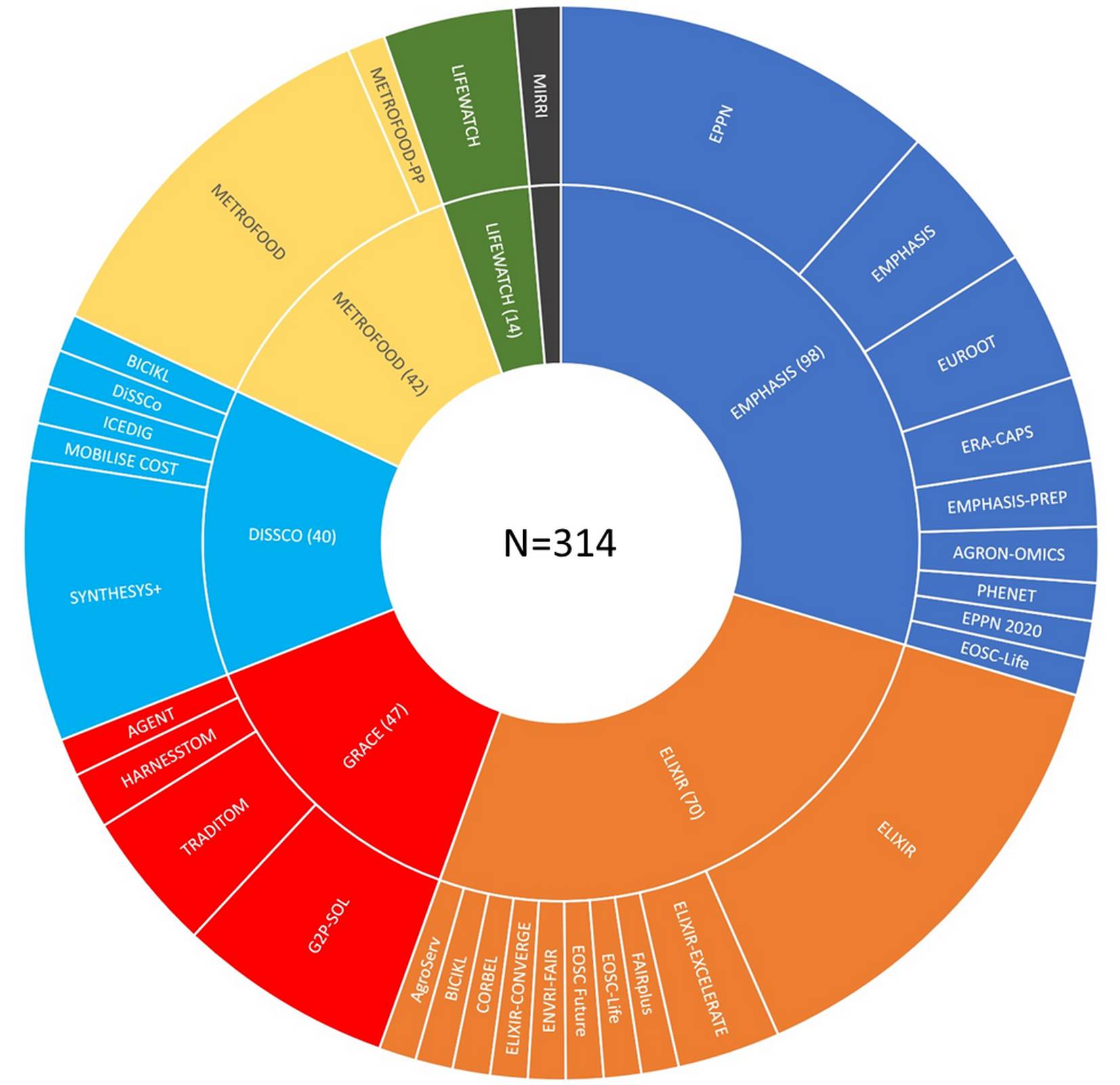


FIG. S2 - Partition of PGR-related papers retrieved from the Clarivate Web of Science™ platform using the name/acronym (outer circle) of the preparatory/collaborative projects considered in this analysis, and their parent RI (inner circle). The overall number of papers retrieved was 314, with largely unequal distribution across the selected RIs.


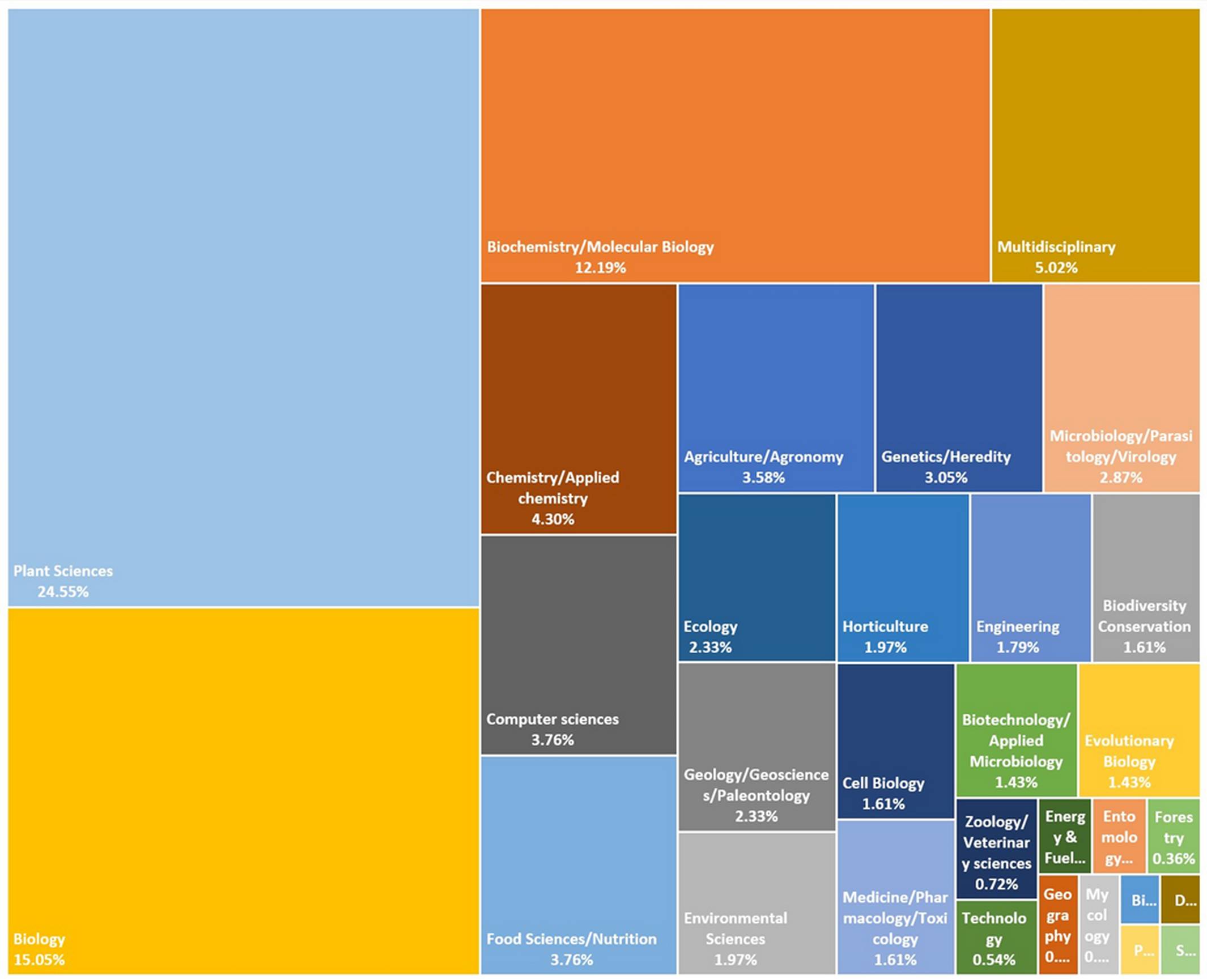


FIG. S3 - Partition of papers (N = 314) by disciplines derived from the WoS categories. To reduce the scattering of data across WoS categories and the heteroscedasticity of the dataset, the 63 distinct WoS categories detected were grouped in 31 large disciplines, which reflected the research areas supported by the 7 RIs included in the analysis.
