# Supplementary material for: A gap and synergy analysis of the European research infrastructure (RI) ecosystem: advancing the novel GRACE-RI dedicated to plant genetic resources
Source: Ann Bot. 2025 Jun 10;136(2):275–85. doi: 10.1093/aob/mcaf092 (PMC12445849; doi:10.1093/aob/mcaf092)
Supplement: mcaf092_Supplementary_Data [file mcaf092_supplementary_data.zip › Supplementary Tables_revised.docx]

# SUPPLEMENTARY TABLES

TAB. S1 - The complete list of the 70 preparatory/collaborative projects analyzed in this study and their parent RIs.

| **Research Infrastructure** | **Project** | **Started** | **Link** |
| --- | --- | --- | --- |
| DiSSCo (5)  www.dissco.eu/ | BICIKL | 2021 | www.bicikl-project.eu/ |
|  | DiSSCo Prepare | 2020 | www.dissco.eu/dissco-ppp/ |
|  | Mobilise COST | 2019 | www.cost.eu/actions/CA17106/ |
|  | ICEDIG | 2018 | www.icedig.info/ |
|  | SYNTHESIS+ | 2019 | www.synthesys.info/ |
| ELIXIR (28)  www.elixir-europe.org/ | AgroServ | 2022 | www.agroserv.eu/ |
|  | B1MG | 2020 | www.b1mg-project.eu/ |
|  | BICIKL | 2021 | www.bicikl-project.eu/ |
|  | Biodiversity Genomics Europe | 2022 | www.biodiversitygenomics.eu/ |
|  | BIOMEDBRIDGES | 2012 | https://elixir-europe.org/about-us/how-funded/eu-projects |
|  | BY-COVID | 2021 | www.by-covid.org/ |
|  | canSERV | 2022 | www.canserv.eu/ |
|  | CINECA | 2019 | www.cineca-project.eu/ |
|  | CORBEL | 2015 | www.corbel-project.eu/ |
|  | EJP RD | 2019 | www.ejprarediseases.org/ |
|  | ELIXIR-CONVERGE | 2020 | https://elixir-europe.org/about-us/how-funded/eu-projects/converge |
|  | EMBRIC | 2015 | www.embrc.eu/ |
|  | EMTRAIN | 2009 | https://elixir-europe.org/about-us/how-funded/eu-projects |
|  | ENVRIplus | 2015 | www.envriplus.eu/ |
|  | EOSC Enhance | 2019 | https://elixir-europe.org/about-us/how-funded/eu-projects |
|  | EOSC Future | 2021 | www.eoscfuture.eu/ |
|  | EOSC4Cancer | 2022 | www.eosc4cancer.eu/ |
|  | EOSC-Life | 2019 | www.eosc-life.eu/ |
|  | EOSCpilot | 2017 | www.eoscpilot.eu/ |
|  | eTRANSAFE | 2017 | https://elixir-europe.org/about-us/how-funded/eu-projects |
|  | EU-STANDS4PM | 2019 | www.eu-stands4pm.eu/ |
|  | EXCELERATE | 2015 | www.elixir-europe.org/excelerate |
|  | FAIRplus | 2019 | www.fairplus-project.eu/ |
|  | HEALTHYCLOUD | 2021 | www.healthycloud.eu/ |
|  | PathOS | 2022 | www.pathos-project.eu/ |
|  | PROPHET | 2011 | www.prophetproject.eu/ |
|  | RI Impact Pathways | 2018 | https://elixir-europe.org/about-us/how-funded/eu-projects |
|  | RItrainPlus | 2021 | https://ritrainplus.eu/ |
| EMPHASIS (11)  https://emphasis.plant-phenotyping.eu/ | AGRON-OMICS | 2006 | https://cordis.europa.eu/project/id/37704 |
|  | CORBEL | 2015 | www.corbel-project.eu/ |
|  | EMPHASIS-GO | 2022 | https://emphasis.plant-phenotyping.eu/infrastructures/cluster-projects/emphasis-go |
|  | EMPHASIS-PREP | 2017 | https://emphasis.plant-phenotyping.eu/about/preparatory-phase |
|  | ENVRIplus | 2015 | www.envriplus.eu/ |
|  | EPPN | 2012 | www.plant-phenotyping-network.eu |
|  | EPPN 2020 | 2017 | https://eppn2020.plant-phenotyping.eu/ |
|  | ERA-CAPS | 2011 | www.eracaps.org/ |
|  | EUROOT | 2012 | www.euroot.eu/ |
|  | PHENET | 2023 | www.phenet.eu/ |
|  | RI-VIS | 2019 | https://ri-vis.eu/network/rivis/home |
| LIFEWATCH (18)  www.lifewatch.eu/ | AgroServ | 2022 | https://agroserv.eu/ |
|  | All-Ready | 2020 | www.all-ready-project.eu/ |
|  | ANERIS | 2023 | https://aneris.eu/ |
|  | BICIKL | 2021 | https://bicikl-project.eu/ |
|  | BIODT | 2022 | https://biodt.eu/ |
|  | DOORS | 2021 | https://www.doorsblacksea.eu/ |
|  | ENVRI-FAIR | 2019 | https://envri.eu/the-envri-fair-project/ |
|  | EOSC Future | 2021 | www.eoscfuture.eu |
|  | EU-LAC ResInfra | 2019 | https://resinfra-eulac.eu/about/ |
|  | FAIR-IMPACT | 2022 | www.fair-impact.eu/ |
|  | MARBEFES | 2022 | https://marbefes.eu/ |
|  | MARCO-BOLO | 2022 | https://marcobolo-project.eu |
|  | Marine SABRES | 2022 | www.marinesabres.eu/ |
|  | OEMC | 2022 | https://earthmonitor.org/ |
|  | PATH2DEA | 2023 | www.path2dea.eu |
|  | PERMAGOV | 2023 | www.permagov.eu/ |
|  | RItrainPlus | 2021 | https://ritrainplus.eu/ |
|  | SUBMERSE | 2023 | https://submerse.eu/ |
| METROFOOD (3)  www.metrofood.com.tr/ | METROFOOD-PP | 2019 | https://www.rivm.nl/en/international-projects/metrofood-pp |
|  | PRO-METROFOOD | 2017 | https://www.rivm.nl/en/international-projects/pro-metrofood |
|  | FNS-Cloud | 2019 | www.fns-cloud.eu/ |
| MIRRI (11)  www.mirri.org/ | AgroServ | 2022 | www.agroserv.eu/ |
|  | BIOINDUSTRY4.0 | 2023 | www.bioindustry4.eu/ |
|  | BY-COVID | 2021 | www.by-covid.org/ |
|  | canSERV | 2022 | www.canserv.eu/ |
|  | CORBEL | 2015 | www.corbel-project.eu/ |
|  | EMBRIC | 2015 | www.embrc.eu/ |
|  | EOSC-Life | 2019 | www.eosc-life.eu/ |
|  | IS_MIRRI21 | 2020 | https://ismirri21.mirri.org/ |
|  | ISIDORE | 2022 | https://isidore-project.eu/ |
|  | MIRRI | 2012 | https://prepphase.mirri.org/home.html |
|  | RI-VIS | 2019 | https://ri-vis.eu/network/rivis/home |
| GRACE (7)  https://www.grace-ri.eu/ | AGENT | 2020 | https://agent-project.eu/ |
|  | BREEDINGVALUE | 2021 | https://breedingvalue.eu/ |
|  | ECOBREED | 2018 | https://ecobreed.eu/ |
|  | G2P-SOL | 2016 | www.g2p-sol.eu/ |
|  | HARNESSTOM | 2020 | http://harnesstom.eu/ |
|  | TRADITOM | 2015 | https://traditom.eu/ |
|  | PROGRACE | 2023 | www.grace-ri.eu/pro-grace |

TAB. S2 - List of the 80 Key Performance Indicators (KPIs) selected to evaluate the activities of the selected RIs supporting PGR research in Europe. (*): derived from D5.2 of the PRO-GRACE project (Guzzon *et al.* 2023).

| **Group** | **No** | **KPI Description** |
| --- | --- | --- |
| GRACE-RI objectives | 1 | Unified information system for both ex situ and in situ conserved PGR |
|  | 2 | Minimum quality standards PGR management |
|  | 3 | Quality certification system for genebanks |
|  | 4 | Standardized methods and descriptors for PGR assessment |
|  | 5 | Ethical, social and regulatory context information on PGR |
|  | 6 | Dissemination, communication and training |
|  | 7 | Multiplication/cultivation/conservation protocols |
| Services* | 8 | Quality management systems (QMS) for PGRFA conservation |
|  | 9 | Safety duplication of germplasm materials |
|  | 10 | Taxonomic validation services |
|  | 11 | Data storage |
|  | 12 | Phenotyping |
|  | 13 | Genotyping |
|  | 14 | Data analysis |
|  | 15 | Phytosanitary aspects and phytosanitary regulation of PGR |
|  | 16 | Policy and regulatory aspects on PGR |
|  | 17 | Education and dissemination |
| Stakeholders* | 18 | CWR genetic reserves |
|  | 19 | Genebanks |
|  | 20 | Farmers |
|  | 21 | Seed companies, crop breeders, etc. |
|  | 22 | Private service providers |
|  | 23 | Other Research Infrastructures |
|  | 24 | Biodiversity Platforms/Data Aggregators |
|  | 25 | Public Research Centers |
|  | 26 | NGOs |
|  | 27 | PGRFA conservation and research network |
|  | 28 | Policymakers, legislators and policy experts |
|  | 29 | Collection Managers, Data curators |
|  | 30 | IT People |
|  | 31 | Institutions |
| Facilities | 32 | Field data collection tools |
|  | 33 | Field stations and field research facilities |
|  | 34 | Seed testing facilities |
|  | 35 | Nutritional analysis laboratories |
|  | 36 | Genotyping laboratories |
|  | 37 | In vitro multiplication facilities |
| Data Types and Data Models | 38 | Passport data |
|  | 39 | Phenotypic/Phenology |
|  | 40 | PGR Infraspecific taxa nomenclature |
|  | 41 | DNA sequences |
|  | 42 | DNA sequence variation |
|  | 43 | Transcriptomics |
|  | 44 | Metabolomics |
|  | 45 | DNA Barcoding/Metabarcoding |
|  | 46 | Ploidy level and cytogenetic analyses |
|  | 47 | Pedigree information and kinship relationships |
|  | 48 | Data on product quality/content of health metabolites, etc. |
|  | 49 | Images of accessions/specimens |
|  | 50 | Agronomic/agricultural data, biotic and abiotic stress, etc. |
|  | 51 | Digital Genetic Objects (DGOs) |
|  | 52 | Metadata and Annotations |
| Data Services | 53 | Ontology cross-mapping |
|  | 54 | Semantic interoperability |
|  | 55 | Taxonomic authorities |
|  | 56 | Enrichment and annotation of data/datasets |
|  | 57 | Data quality checks / Minimum quality standards |
|  | 58 | Method and data standardization |
|  | 59 | Loans & visit system |
|  | 60 | Data best practice and guidance |
|  | 61 | Digital Library |
|  | 62 | Digital Collection List/Registry |
|  | 63 | Catalogue of Tools |
|  | 64 | Dataset Repository |
|  | 65 | Dataset Access and Data Management |
|  | 66 | API (Application Programming Interfaces) |
|  | 67 | Data analysis and visualization tools |
|  | 68 | Modelling software platforms |
|  | 69 | Virtual research environment |
|  | 70 | Data transfer protocols and schemas |
|  | 71 | Data conversion middleware |
|  | 72 | Database crawling and harvesting |
|  | 73 | Data usage statistics |
| Other activities | 74 | Public education and awareness |
|  | 75 | Knowledge base |
|  | 76 | Public challenges and competition (gamification) |
|  | 77 | Storytelling |
|  | 78 | Courses, workshop and e-learning |
|  | 79 | Academic programmes |
|  | 80 | Consulting and expertise |

# LITERATURE CITED

**Guzzon F, Maggioni L, Goritschnig S, et al. 2023**. Identification of the scientific services, stakeholders, promoters, and utilizers of the proposed RI (version 1). PRO-GRACE project. https://www.grace-ri.eu/fileadmin/user_upload/Pro-grace/Img/Deliverables/D5.2.pdf. 29 Apr. 2025.
