# Supplementary material for: A gap and synergy analysis of the European research infrastructure (RI) ecosystem: advancing the novel GRACE-RI dedicated to plant genetic resources
Source: Ann Bot. 2025 Jun 10;136(2):275–85. doi: 10.1093/aob/mcaf092 (PMC12445849; doi:10.1093/aob/mcaf092)
Supplement: mcaf092_Supplementary_Data [file mcaf092_supplementary_data.zip › aob-25033-s01.docx]

Dear Editor,

I am pleased to submit the revised version of the manuscript 25033 entitled “A gap and synergy analysis of the European Research Infrastructure (RI) ecosystem: advancing the novel GRACE-RI dedicated to Plant Genetic Resources” for consideration in Annals of Botany as a Perspective paper. The new version takes into account the suggestions of the Handling Editor and the Reviewer, below is a detailed description of all the changes answering point by point. As requested, a 'track changes' version of the manuscript is provided as a Word file as 'supplementary material'.

We believe that the review process has improved the manuscript and made it more accessible to all potential readers interested in the topic of PGR.

We thank you for your time and attention. Should you require any further information, please do not hesitate to contact me.

Best regards,

Domenico De Paola on behalf of all authors

**Answers to the handling editor's comments:**

1) With respect to GRACE-RI itself, a brief summary of what this program includes and how this dovetails with other programs would be useful for readers who are not overly familiar with European programs and are interested in genebank conservation and germplasm use in breeding. This would be most useful on page 9 between lines 24 and 25, or sometime earlier, so that when the intersection of these programs is discussed in the gap and synergy analysis, the reader already knows what GRACE-RI entails (this is not clear at the moment, at least to someone like myself not overly familiar with the current frameworks). Additionally, in the analysis, GRACE-RI is used as a proxy but it is unclear in the main text what special parameters are given to the proposed program that make it different or complementary to current programs (generalities are given that are used for the comparison but they are too general for a nonspecialist). One way to address this point is to move up the 'GRACE-RI: A new pillar for the PGR community' section. In this way, the program can be introduced and specifics of what this program will address that is currently lacking (and/or how the program will bridge other programs) can be explained in advance of the comparisons. In this way the analysis can be used as a support to show that GRACE can fill the niches identified. It is also unclear from the manuscript whether GRACE is being proposed or is already in progress and this should be clarified. (NB. The final paragraph in 'GRACE-RI: A new pillar for the PGR community' that discusses links between GRACE, ELIXIR, and METROFOOD is still more suitable to a discussion after the analysis.). In summary, please do be specific about how/what would need implementing in GRACE in a way that is as widely accessible as possible. It would be helpful to have the description of GRACE set up so that it complements the information in the figures by describing how it differs/strengthens (a) research aims (b) research products (c) facilities services relative to other programs.

In response to your feedback, we have revised the manuscript to enhance both its logical flow and clarity. Specifically, we have moved the section ‘GRACE-RI: A New Pillar for the PGR Community' before the section on landscape analysis (page 9, line 4). In this paragraph, an introductory statement has also been added, to make the aims and potential of the proposed RI more accessible to all readers interested in germplasm conservation and use (page 9, lines 5-16). This revision better highlights the project objectives and the potential services that GRACE-RI can offer to the PGR community. We believe that the framework within which the gaps and synergies between GRACE-RI and existing European infrastructures are assessed is now clearer, as is the relation of GRACE-RI to other existing RIs. Regarding the implementation status of GRACE-RI, the manuscript now clarifies that it is currently a proposal of the authors, who are representatives of the PRO-GRACE consortium, and the RI assessed in the gap and synergy analysis (page 12, lines 12-15).

2) Please use consistent UK spelling.

Thank you for your comment. We reviewed the manuscript and ensured that UK spelling has been used consistently throughout.

3) "By doing so, it could help harmonizing and standardizing the European PGR research landscape and reducing data fragmentation by addressing the existing gaps." Should read: Additionally, doing so could help harmonize and standardize the European PGR research landscape and reduce data fragmentation by addressing the existing gaps.

This sentence has been deleted in the revised version.

4) "Linking genotypic and phenotypic data can help exploiting core" Should read:Linking genotypic and phenotypic data can help exploit core

As requested, we revised the sentence (page 7, line 14).

5) You write: "maintaining the evolutionary potential of PGR" but it seems more correct that this is the breeding potential of PGR.

Thank you for the suggestion. We have incorporated the phrase *"useful to breeding"* into the revised sentence (page 7, line 17).

6) Figure captions:
I. Fig1 CORDIS-EU FS - what is FS? Please expand RI, WoS, KPI (in first instance). Please say what the reference Blondel et al. 2008 is for i.e., is it a method? I think the method is referenced with the same reference in the main text and so the ref is not needed here.

Definitions for the acronyms RI, FS, WoS, and KPI have been provided in the caption of the Figures in the manuscript (Pag. 28). In accordance with your suggestion, we have removed the Blondel reference from the figure caption.

II. Fig2, Fig3 Expand RI, FS, WoS, KPI in first instance.

Definitions for the acronyms RI, FS, WoS, and KPI have been provided in the caption of the Figures in the manuscript (Pag. 28).

7) Figures:
   Fig2 - (a) PC1 axis has the numbers upside down. One axis has PC2 upside down. Please correct the letter/number orientations.

Thanks for noticing. We revised FIG. 2 according to your suggestions.

Fig2 - (b)PC1 axis is cut off. Can PC2 be made more legible?

Thanks for noticing. We revised FIG. 2 according to your suggestions.

.

**Answers to the reviewer comments:**

Referee's Review for 25033:

1) The authors needs to more clearly identify themselves: throughout the paper, the positioning of the authors is unclear: from whom and for what are the authors speaking, are they representative of the entire PRO_GRACE or GRACE-RI consortium? Are they legitimate to defend this project? I am convinced so, but it needs to be written down for the readers.

In the revised version of the manuscript, we have added a sentence clarifying the authors' roles within the PRO-GRACE project consortium and the research infrastructures included in the gap and synergies analysis (page 12, lines 12-15).

2) What is an ERI, ESFRI and the scope of the other existing ERI mentioned? An additional separate Box for example would be necessary.

We appreciate your suggestions and have revised the manuscript accordingly to include the following phrase: " The above scenario emphasises the need for a coherent European policy framework and the development of a long-term European RI dedicated to PGR, encompassing large-scale facilities, digital platforms and essential services " (page 6, lines 4-6), this better explain what is an RI in the context of the General Overview.

We have also provided a more precise definition of the European Strategy Forum on Research Infrastructures (ESFRI) (page 6, lines 7-9) and clarified the importance of the ESFRI Roadmap as a key strategic tool (page 6, lines 12-13). The Table S1 now contains a direct link to the official websites of the other existing ERI mentioned; the corresponding domain and scope have been provided in the main (page 12, lines 16-21)

3) In the general overview, the authors also need to improve their description of the current state of PGR conservation in Europe: how many genebanks exist, how many initiatives, and accessions, how heterogenous is the field? To which extent existing elements (ECPGR) are not already some kind of informal ERI ? what is at stake here for the field?

In line with the title of the section "General Overview of PGR in Europe," we have strengthened the focus on the European network of genebanks whose accessions are currently listed in the EURISCO catalogue, providing specific information on the number of participating institutions (page 4, lines 14-17).

Additionally, we emphasise the role of ECPGR (a key partner of PRO-GRACE), but also stress the need for a European RI dedicated to PGR, encompassing large-scale facilities, digital platforms and essential services (page 5, lines 21-25; page 6 lines 1-6).

4) The exact scope of the GRACE-RI is also to be precise: it sounds like the initiative is mostly shaped for genebanks, i.e for ex situ conservation, letting alone the ways or means by which the new ERI must be coordinated to 1) global effort for in situ conservation, in particular related to CWR that are on the "grey zone" between crops and wild plants, 2) coordination with many initiatives that try to facilitate on farm conservation... in short, anything that is actually -not- genebank but still performing in the PGR field.

In the revised section “GRACE-RI: A New Pillar for the PGR Community*”*, we clarified that GRACE-RI could strengthen the implementation of *in situ* and on-farm conservation of plant genetic resources (PGR) as complementary approaches to the *ex situ* conservation, while promoting a broader and more coordinated network of conservation centres (page 9, lines 5-17).

5) Eventually, the issue with such an ERI is probably to keep every actors at the same page. The genebank field is very heterogenous in Europe, and the big one will probably be reluctant to be in such an ERI without substantial advantages, and the smalle one desperate to participate. How does the implementation of the ERI -internally- is planned?

As the reviewer noted, the PGR sector is highly diverse, encompassing not only genebanks but also *in situ* and on-farm conservation, and involving a wide range of stakeholders such as policymakers, farmers, breeders, and researchers. To accommodate this diversity in motivations, capacities, and resources, a tiered participation model is being considered. This would enable institutions to engage at levels aligned with their strategic goals and means. Incentives include access to shared services, harmonized standards, increased visibility in the European research landscape, and support for capacity building, as detailed in the manuscript. Larger institutions could provide strategic leadership, while smaller or underfunded ones could benefit from shared infrastructure and services. The approach seeks to balance national autonomy with collective European goals, ensuring that all actors see clear value in contributing to a common PGR infrastructure. In line with the principle that plant genetic resources are a “common heritage of mankind,” long-term genetic diversity conservation and food security depend on broad collaboration—no single institution can succeed alone. Even small centres may hold unique accessions of great value to the wider plant research community. The GRACE-RI consortium can build on the 40+ years of experience of the European Cooperative Programme for Plant Genetic Resources (ECPGR) in fostering effective conservation and sustainable use across Europe.

6) Figure 3 might actually be quite redundant with the two other figures and therefore not really needed.

Whilst we appreciate the comment, we believe that FIG. 3 can help to clarify the degree of correlation between the RIs.
